# A model for specific goals for in-patient treatment linked to resources and limitations in out-patient treatment

**DOI:** 10.1192/bjb.2020.31

**Published:** 2020-12

**Authors:** Virginia Davies

**Affiliations:** South London and Maudsley Mental Health NHS Trust, UK

**Keywords:** In-patient treatment, child psychiatry, alternative models, Norwegian comparisons, front door expertise

## Abstract

The author reflects on the Norwegian inpatient service descriptions contained in Wilkinson's article, considering the challenges laid out in his piece from the perspective of a child and adolescent psychiatrist working in a hospital crisis setting, as well as within the context of child and adolescent mental healthcare staffing across the UK.

As a paediatric liaison psychiatrist, I was dubious about how an article that concerns itself with goals for in-patient care would speak to my own experiences and practice. I need not have worried; Wilkinson's paper^1^ is rich in reflections about the core issues at play in child and adolescent psychiatric practice and has relevance for all child and adolescent psychiatrists.

Wilkinson writes from the vantage points of retirement and from being ‘over the water’. Having spent his working life practising in the well-funded healthcare system of Norway, he brings a huge amount of digested clinical encounter to his analysis of how in-patient services are most usefully oriented, around safety and the developing personality. He argues for two different types of in-patient unit, which might be considered as future service development objectives within the UK. Both rely on considerable expertise and stable staffing. His ‘safety first’ model requires most child and adolescent psychiatric expertise at the emergency and out-patient levels, with less direct psychiatric input during in-patient admission. He acknowledges that we are a long way from a point where we might manage this, but how far off are we?

In the UK, the number of consultant child and adolescent psychiatrists per head of child population is significantly, perhaps scandalously, lower than for other specialties. As of September 2019, provision ranged from 2.8 consultant child and adolescent psychiatrists per 100 000 children in Kent, Surrey and Sussex to a high of 10.7 per 100 000 children in North Central and East London. This compares with 29.3 consultant paediatricians per 100 000 children across England. Most UK general hospitals do not have not a single consultant child and adolescent psychiatrist in their employ. Accordingly, the majority of emergency departments catering for under-18s have no departmental consultant child and adolescent psychiatrist either.

Wilkinson's powerful charge really resonated with me for this reason: ‘Why is it not expected that A&E departments should be catering for the second highest cause of mortality?’ It is clear what he means by ‘catering for’; he has earlier described using in-house ‘child and adolescent psychiatry expertise’.

He notes the value of front door expertise: ‘those with greater experience refer fewer patients for admission because of potential suicide’. Experienced staff also take less time to come to a decision and tend to provide greater psychological containment, be this in the care of a child with a perturbing mental health presentation or one with a serious physical ailment. Junior psychiatrists, emergency department staff of all grades, and patients and their families all know this. So why are we not stacking more experience to the fore as per Wilkinson's ‘safety first’ model?

In discussing why local in-patient work is so vital for the ‘safety first’ cohort, Wilkinson describes the task thus: ‘It is not the axis 1 diagnosis which determines the need for admission, but the necessity of an alternative “containment” of the patient to that available at home. Two factors operate here: the state of the child and the processes at home.’ Working in an emergency setting, a very large proportion of those we refer for admission fall into the second bracket. This is the reason I have watched the stripping back of family therapy provision within in-patient settings with a deal of dismay.

Wilkinson highlights the need for high and stable levels of staffing in the ‘intensive’ (24/7) units needed for the ‘safety first’ cohort. This brings us back to the central importance in recovery at any state or stage of relationships and attachment. How many of our child and adolescent in-patient units have ‘a stable group of nurse therapists’? How many even have enough staff each shift? He comments on the reduced need for restraint and sedation in Norwegian units, an enviable outcome of such stable staffing.

Going on to consider the needs of a second cohort of patients, he describes how counterproductive it is to admit those with developing personality issues to the same type of unit as that used for the ‘safety first’ cohort. He proposes instead a weekday unit, with greater direct involvement by psychiatry, who are less directly involved in day-to-day patient care in the 24/7 ‘intensive’ units. The benefit of a 5-day unit is the testing out and information gathering that can be garnered by weekends at home.

In the UK, psychiatric admission for those with personality issues is generally to the same unit as the ‘safety first’ cohort. The idea of the 24/5 unit, with its strong emphasis on attachment, trauma and loss, sounded like an extension of what our current dialectical behaviour therapy teams offer, but with the added containment for the rest of the system of not having responsibility for the child or young person during their most risky stages. Our ‘ordinary’ in-patient units already focus on axes 2–5. And I don't think most in-patient settings in their current form are failing to consider trauma and loss. But staffing issues are the real ‘elephant in the room’. We have so much nursing ‘churn’ and such a paucity of senior staff capacity that both the intensive 24/7 unit model, with its reliance on stable nursing staff and high-level emergency and out-patient expertise, and the 24/5 ‘developing personality’ unit model, with its reliance on intensive consultation and liaison with other agencies, especially social care, feel like impossible dreams for us here in the UK.

It would be easy to close by asking ‘Please can I move to Norway?’, but I suppose the challenge laid down to us in this paper is this: what can we do individually as citizens, or as a professional collective, to try to address this most gross injustice of utterly inadequate mental health services, emergency, out-patient and in-patient, for some of the most vulnerable members of our society?

## About the author

**Virginia Davies**, MRCP, MRCGP, FRCPsych, is a Consultant in Paediatric Liaison in the South London and Maudsley National & Specialist CAMHS Paediatric Liaison Service at King's College Hospital Denmark Hill, London, UK.
