# A model for specific goals for in-patient treatment linked to resources and limitations in out-patient treatment – ERRATUM

**DOI:** 10.1192/bjb.2020.142

**Published:** 2022-06

**Authors:** Virginia Davies

In the original published article, the country name was missing from the author's affiliation. This has now been corrected.
